# Structural basis of human α7 nicotinic acetylcholine receptor activation

**DOI:** 10.1038/s41422-021-00509-6

**Published:** 2021-05-06

**Authors:** Yue Zhao, Sanling Liu, Yingxin Zhou, Mengge Zhang, Haopeng Chen, H. Eric Xu, Demeng Sun, Lei Liu, Changlin Tian

**Affiliations:** 1 Hefei National Laboratory of Physical Sciences at Microscale, Anhui Laboratory of Advanced Photonic Science and Technology, and School of Life Sciences, University of Science and Technology of China, Hefei, Anhui China; 2 Shanghai Institute of Materia Medica, Chinese Academy of Sciences, Shanghai, China; 3 Tsinghua-Peking Joint Center for Life Sciences, Ministry of Education Key Laboratory of Bioorganic Phosphorus Chemistry and Chemical Biology, Department of Chemistry, Tsinghua University, Beijing, China; 4 High Magnetic Field Laboratory, Chinese Academy of Sciences, Hefei, Anhui China

**Keywords:** Cryoelectron microscopy, Ion channel signalling

Dear Editor,

Nicotinic acetylcholine receptors (nAChRs) are a class of pentameric ligand-gated ion channels (pLGICs) widely expressed in nervous system. nAChRs function as neurotransmitter receptors that respond to endogenous acetylcholine and choline, modulating neuronal excitability and synaptic communication. The homomeric α7 nAChR is among the most abundant subtypes of nAChR in the brain. Dysfunction of α7 is found to be associated with several neuropsychiatric and neurologic disorders, including schizophrenia and Alzheimer’s disease.^1,2^ Stimulation of α7 has been reported to improve attention, cognitive performance, and neuronal resistance to injury. Therefore, agonists and positive allosteric modulators (PAMs) of α7 have become hot candidates in the drug development for the treatment of α7-related diseases.^3,4^ EVP-6124 (abbreviated as EVP) is a high-affinity α7-selective agonist.^5^ PNU-120596 (abbreviated as PNU) is the first reported α7-selective PAM that could increase the peak current of the receptor evoked by agonists and delay channel desensitization.^6^ Both EVP and PNU are in clinical trials for the treatment of Alzheimer’s disease, schizophrenia, and cognitive impairment. Despite the significance of α7 in physiology and pharmacology, the mechanisms underlying the activation of α7 upon agonist and/or PAM binding remain elusive. Little is known about the structural basis of the higher selectivity of EVP and PNU for α7, which would be highly valuable for rational drug development targeting the receptor. Herein, we report the structures of full-length human α7 in apo, EVP-bound and EVP/PNU-bound states at 3.18, 2.85 and 3.02 Å, respectively (Fig. 1a–c; Supplementary information, Figs. S1–S4 and Table S1).

**Fig. 1:**
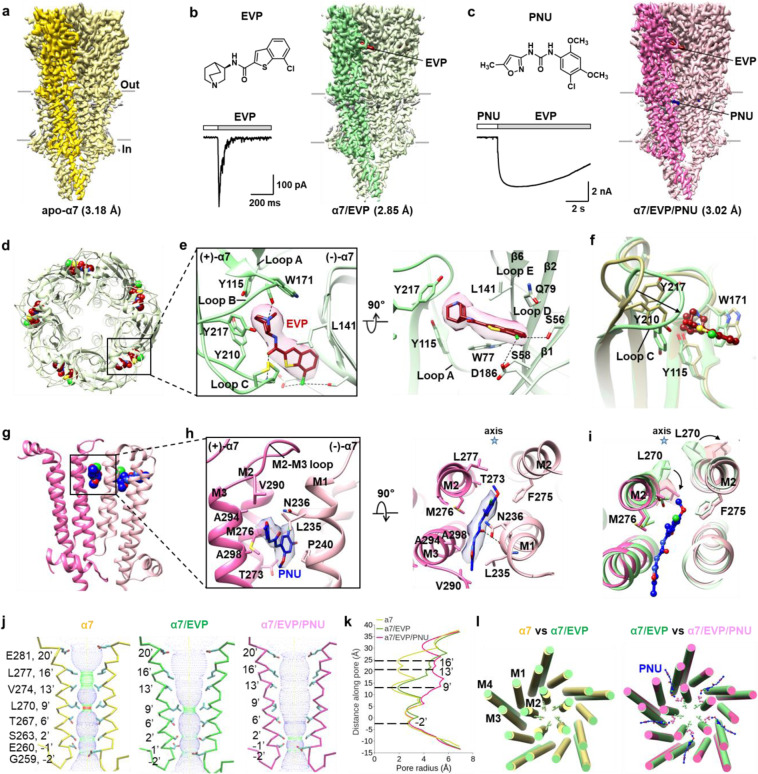
Cryo-EM structures of human α7 nicotinic receptor in different states. **a**–**c** Cryo-EM maps of human α7 in apo (**a**), EVP-bound (**b**) and EVP/PNU-bound (**c**) states. Chemical structures of EVP and PNU are shown. Representative responses of α7 are shown for the application of 20 µM EVP (**b**), and pre-application of 10 µM PNU followed by 20 µM EVP (**c**). **d** Overview of the binding sites for EVP from the extracellular space. EVPs are shown as spheres. **e** Molecular details of the EVP-binding interface viewed from the extracellular space (left) and parallel to the membrane plane (right). EVP is shown as sticks with corresponding density. **f** Conformational changes in the ligand-binding pocket. Structures of apo-form and EVP-bound α7 are represented as yellow and green ribbons, respectively. **g** Overview of the binding sites for PNU. PNU is shown as spheres. **h** Molecular details of the PNU-binding interface viewed parallel to the membrane plane (left) and from the extracellular space (right). PNU is shown as sticks with corresponding density. **i** Conformational changes in the PNU-binding pocket are shown from the extracellular space. Structures of EVP-bound and EVP/PNU-bound α7 are represented as green and pink ribbons, respectively. Densities for L270 in both the α7/EVP and α7/EVP/PNU models are shown. **j** Two M2 helices are shown for each of the conformational states. Side chains of pore-lining residues are shown. The channel pore radius was calculated using the HOLE program (red < 1.5 Å ≤ green ≤ 3.0 Å < blue). **k** Plots of pore radius for α7 in different states along the pore axis. The α-carbon position of 0′ Lys (Lys261) is set to zero. **l** Comparisons between apo (yellow) and EVP-bound α7 (green) (left), EVP-bound (green) and EVP/PNU-bound α7 (pink) (right), viewed from the extracellular space. Side chains of L270 are shown in ball and stick representation.

The apo-form structure, representing a resting state of α7, has an overall structure similar to that of other nAChRs.^7–10^ Five identical subunits surround a central pore axis with 5-fold symmetry, forming a typical pentameric assembly. Each α7 subunit comprises an N-terminal extracellular domain (ECD), a C-terminal transmembrane domain (TMD) and an intracellular domain (ICD) (Supplementary information, Fig. S5a, b). Glycosylation sites (Asn46 and Asn133) are present in all subunits. When this manuscript is in preparation, Noviello et al. reported cryo-EM structures of human α7 in antagonist (α-bungarotoxin, α-bgt)-bound closed state, agonist (Epibatidine)-bound desensitized state and agonist (Epibatidine)/PAM (PNU)-bound activated state.^11^ The overall structures of apo and α-bgt-bound α7 are almost the same (Supplementary information, Fig. S5c). In particular, the pore profile of apo-form α7 reported here is similar to that of α7 in α-bgt-bound state (Supplementary information, Fig. S5d). The narrowest point in the pore is located at L270 lying at the 9′ position in M2 helix, with a radius of ~1.4 Å, which is too narrow for the passage of a hydrated cation, suggesting a closed conformation of the channel. This is consistent with previous inference that the 9′-Leu gate, formed by side chains oriented towards the pore axis, is generally conserved in the Cys-loop receptor structures in α-bgt-bound closed states, as well as in apo-resting states.^7^

In the structure of α7/EVP complex, five EVP molecules in total are observed, each of which binds at the typical neurotransmitter binding site located between the ECDs of two adjacent subunits (Fig. 1b, d). The quinuclidine group in the head of EVP is enclosed by an aromatic cage formed by residues Y115, W171, Y210 and Y217 in the (+)-subunit and W77 and L141 in the (–)-subunit. The basic bridging amino nitrogen of EVP makes cation–π interactions with W171, Y210 and Y217 and hydrogen bond interactions with the carbonyl oxygen of W171 (Fig. 1e). The carbonyl oxygen of EVP forms a hydrogen bond with the amide nitrogen of C212. Notably, the benzothiophene group in the tail of EVP stretches out of the aromatic cage, inserting into a hydrophilic pocket formed by S56, S58, Q79, Q183 and D186 in the (–)-subunit and E211 in the (+)-subunit (Fig. 1e).

The overall structures of α7 in α7/EVP and α7/Epibatidine complexes show high similarity (Supplementary information, Fig. S6a). EVP binds at the orthosteric site in close proximity to that in the α7/Epibatidine structure, while it has an orientation strikingly different from that of Epibatidine (Supplementary information, Fig. S6b). The position of the quinuclidine head moiety of EVP considerably overlaps that of Epibatidine, while the orientation of the benzothiophene group of EVP and the chloropyridine group of Epibatidine form an angle of ~90° (Supplementary information, Fig. S6b).

The structure of α7/EVP complex provides insights into ligand selectivity between nAChR subtypes. Sequence alignments and structural comparisons of EVP-bound α7 with nicotine-bound α4β2- or α3β4-nAChR reveal that the aromatic cages warping these different ligands contain conserved residues and have highly similar shapes, suggesting that the cage makes little contribution to the agonist selectivity (Supplementary information, Fig. S7a). By contrast, the complementary faces in the (–)-subunits are divergent. In particular, two substitutions in the binding pocket are S56 (M36 in β2 and Q38 in β4) and Q79 (T59 in β2 and K61 in β4) in loop D of α7. Superimpositions reveal potential clashes of the benzothiophene group on EVP with M36 in β2 and Q38 in β4 (Supplementary information, Fig. S7b, c). Our FLIPR (Fluorescence Image Plate Reader) and calcium flux measurement data showed that the affinity of mutants S56Q, Q79T and Q79K for EVP are decreased compared to that of wild-type α7 (Supplementary information, Fig. S7d), consistent with previous mutation-based functional studies. These studies indicate that the selectivity of EVP to α7 must be highly correlated with the complementary subunit of the pentameric channel.

The structures of α7 in apo and EVP-bound states allow us to investigate the conformational changes of the receptor upon agonist binding. When the ECDs of apo-form and EVP-bound α7 are superposed, prominent conformational changes are observed in the ligand-binding pocket (Fig. 1f). Loop C undergoes a striking inward flip. The Cα atoms of C212 at the tip of loop C shift by 7.1 Å. The flipping of loop C gives the α7/EVP complex a remarkably cramped binding pocket compared to that in the apo form. The displacement of W171 from loop B and ~180° rotation of Y115 from loop A are observed, resulting in formation of an aromatic cage for the ligand. In the TMD, the prominent conformational change is the relocation of M2 helix (Supplementary information, Fig. S8). The upper half of M2 undergoes both upward movement and outward rotation, while the lower half displays a slight upward movement. By contrast, the packing of M1, M3 and M4 helices remains similar in the structures of apo-form and EVP-bound α7. These cause the residues lining the pore in the upper half of M2 to move away from the channel axis, opening the upper half of the pore. Moreover, the post-M3 loop undergoes an upward and outward movement away from the lateral portals in the structure of EVP-bound α7. We propose that the conformational changes in the post-M3 loop observed in EVP-bound α7 structure are related to channel opening, as concluded in the structural studies of the 5-HT_3A_ receptor.^12^

In the EVP/PNU-bound α7 structure reported here, PNU is unambiguously assigned and found to insert into the TMD interface of two adjacent subunits (Fig. 1c, g). The observed PNU binding site in our structure is different from the previously reported cavity surrounded by the four transmembrane helices of α7 found through a “blind docking” approach.^13^ PNU interacts with hydrophobic residues in M2 and M3 of the (+)-subunit and in M1 and M2 of the (–)-subunit (Fig. 1h). Specifically, the larger halogenated aromatic group of PNU is in close proximity to T273, M276 and L277 in (+)-M2 and F275 in (–)-M2. Meanwhile, its isoxazole heterocyclic group points outward, interacting with V290 from the M2–M3 loop, A294 and A298 from M3, and L235, N236 and P240 from M1. A hydrogen bond between the carbonyl of PNU and the carbonyl oxygen of L235 probably provides an additional stabilizing interaction (Fig. 1h). Our structure data are consistent with previous mutation analysis, which revealed that F275, M301, M276 and N236 were important for allosteric modulation by PNU. Notably, despite including a high concentration of PNU in the cryo-EM sample, PNU molecules were not confidently positioned in the Epibatidine/PNU-bound α7 structure reported by Noviello et al. It was suspected that the locally dynamic nature of the TMD in the activated state, which correlates with its lower local resolution, precludes visualization of the small molecules.^11^

Aiming to investigate the structural basis for the selectivity of PNU to α7, we compared the structures of the TMDs from EVP/PNU-bound α7 and from nicotine-bound α4β2- or α3β4-nAChR. By superimposing TMDs in the (+)-subunit, several notable structural differences in regions in proximity to the PNU binding site were found (Supplementary information, Fig. S9). First, the upper half of M1 in both α4β2- and α3β4-nAChR shows slight shift towards M3, resulting in a much narrower cleft that is not sufficient to accommodate the isoxazole heterocyclic group of PNU, which would clash with both I214 in β2 and I216 in β4. Second, A298 in M3 is replaced by I285 and I278 in the α4 and α3 subtypes, respectively. The larger side chain of isoleucine could cause steric hindrance of PNU binding. Third, M276 in M2 is replaced by L263 in α4 and L256 in α3. Since the side chain of leucine is much more rigid than that of methionine, the compatibility of leucine in the PAM-binding pocket is reduced. This is consistent with the previous observation that the M276L substitution strongly reduced the PNU sensitivity of human α7.^13^

Superimposition of EVP-bound and EVP/PNU-bound α7 structures shows that the binding of PNU causes a notable change at L270, which rotates away from the pore axis by ~110° (Fig. 1i). L270 separates M2 into upper and lower halves, and the upper part also displays a slight clockwise rotation. These rotations expand the channel pore near L270 and the upper part in the PNU-bound α7, which is in contrast to almost identical structures in the ECD, the ICD and the remaining regions of the TMD in the EVP- and EVP/PNU-bound states. By contrast, in the Epibatidine/PNU-bound α7 structure, the side chain of L270 is not well ordered.

Inspection of α7 structures in three states (apo, EVP-bound, EVP/PNU-bound) reveals four notable constriction sites within the ion channel pore: 16′-Leu (L277), 13′-Val (V274), 9′-Leu (L270) and –2′-Gly (G259) (Fig. 1j, k; Supplementary information, Fig. S10). Estimates of the cross-sectional area of the pore from the distance between adjacent Cα atoms at the 16′-, 13′- and 9′-positions show an ascending order from the apo to the EVP-bound state and then the EVP/PNU-bound state. In contrast, the distances between the –2′ positions remain almost constant. The apo-form structure of α7 has the narrowest ion channel with a radius of ~1.4 Å at 9′ position (Fig. 1k). In the EVP-bound form, the upper half of M2 rotates away from the 5-fold axis, expanding the pore radii at the 16′ and 13′ positions from ~1.9 Å to ~4.0–4.5 Å (Fig. 1k). 9′ position undergoes much less expansion than 16′ and 13′ positions, thus retaining a pore constriction site with a radius of ~1.9 Å. In the EVP/PNU-bound structure, the further rotation of L270 (9′ position) side chain leads to a marked dilation of the upper half of the pore, leaving the region around –2′ position as the only remaining constriction section (Fig. 1k).

Interestingly, structural comparison of EVP/PNU-bound α7 with Epibatidine/PNU-bound α7 reported by Noviello et al. reveals a striking divergence in the pore profile (Supplementary information, Fig. S11). In the Epibatidine/PNU-bound α7, the diameter of the transmembrane pore near 9′-Leu increases to 7.2 Å, representing an open conformation of the channel pore. α7 is known to undergo desensitization on the millisecond time scale upon agonist binding; thus α7/EVP structure is suggested to be in desensitized state. The EVP/PNU-bound α7 displays a wider pore than the EVP-bound form but narrower pore than the Epibatidine/PNU-bound form, suggesting that it represents a partially desensitized or partially open state. The pore at 9′ position in the EVP/PNU-bound α7 may be contributed by the pore stabilization by PNU, and consequently related to the enhanced cation conductance followed by slow decay. It has been found that the mutation of Leu at 9′ position to Thr causes a dramatic loss of desensitization of the current and prolongs the conducting state at the single-channel level, probably by reducing the hydrophobic barrier of the ion conductance pore.^14^ Therefore, we propose that Leu at 9′ position is the critical site that determines the desensitization of nAChRs (Fig. 1l).

In conclusion, we present cryo-EM structures of the human α7 nicotinic receptor in apo-resting, agonist-bound desensitized, and agonist/PAM-bound partially desensitized states, providing insights into the conformational changes involved in α7 activation. More importantly, our structures revealed detailed structural basis for the binding of α7-selective agonist EVP-6124 and PAM PNU-120596, illuminating the structure mechanism underlying selectivity of these drug candidates for α7. All of the above findings provide a framework for probing α7’s unique biophysical and pharmacological properties, and will assist rational drug development to activate α7 and alleviate neurological disorders.

## Supplementary information

Supplementary information

## Data Availability

Cryo-EM maps of apo-form α7, α7/EVP complex and α7/EVP/PNU complex have been deposited in the Electron Microscopy Data Bank (http://www.ebi.ac.uk/pdbe/emdb/) under accession codes: EMDB-31168, EMDB-31172 and EMDB-31176, respectively. The corresponding atomic coordinates have been deposited in the Protein Data Bank (http://www.rcsb.org/pdb) under accession codes: 7EKI, 7EKP, and 7EKT, respectively.
