# Supplementary material for: Structural basis of human α7 nicotinic acetylcholine receptor activation
Source: Cell Res. 2021 May 6;31(6):713–6. doi: 10.1038/s41422-021-00509-6 (PMC8169775; doi:10.1038/s41422-021-00509-6)
Supplement: Supplementary file 1 — Supplementary information [file 41422_2021_509_MOESM1_ESM.pdf]

Supplementary information

Methods and Materials

Cloning and protein expression

Genes were synthesized by Genescript. The sequence of enhanced GFP was inserted into the loop between helices MX and MA of human α7 nAChR (Uniprot: P36544) to produce $\alpha7_{EM}$. DNA encoding $\alpha7_{EM}$ and human NACHO (Uniprot: Q53FP2)[1] were cloned into a pcDNA3.1 vector separately, with a FLAG tag (DYKDDDDK) at the carboxyl terminus of $\alpha7_{EM}$. For protein expression, HEK293F cells were cultured at 37 °C and 5% CO_2 in SMM-293TI medium (Sino Biological Inc.) and were used for transfection at a density of 2×10^6 cells/mL. Plasmids for $\alpha7_{EM}$ and NACHO were co-transfected into HEK293F cells using PEI reagent. Transfected cells were cultured for 60 h before harvesting.

Protein purification

Cell pellet from 1 L of culture was resuspended in lysis buffer (150 mM NaCl, 10% Glycerol, 20 mM Tris, pH 8.0). The suspension was supplemented with 1.5% (w/v) n-dodecyl-β-D-maltopyranoside (DDM, Anatrace), 0.3% (w/v) cholesteryl hemisuccinate (CHS, Sigma) and protease inhibitor cocktail (APExBIO). After incubation at 4 °C for 2 h, the insoluble fraction was removed by ultra-centrifugation at 180,000× *g* for 45 min at 4 °C and the supernatant was incubated with anti-Flag M2 affinity resin (Sigma) at 4 °C. The resin was then collected and washed with wash (W) buffer (150 mM NaCl, 0.06% GDN, 20 mM Tris, pH 8.0). The proteins were eluted with W buffer supplemented with 200 μg/mL FLAG peptide. After elution, the proteins were concentrated and further purified on a Superose 6 increase column (GE healthcare) equilibrated with 150 mM NaCl, 0.02% GDN, and 20 mM Tris, pH 8.0. Peak fractions of α7 protein were collected and concentrated to ~4 mg/mL for cryo-EM experiments. For the sample preparation of different α7 complexes, a final concentration of 100 μM

EVP-6124, or 100 μM EVP-6124 and 100 μM PNU-120596 were added into the protein, respectively, and incubated for 1 h before cryo-EM sample verification.

Cryo-EM sample preparation and data acquisition

A total of 3 μL of protein sample at ~4 mg/mL was applied to freshly plasma-cleaned (H_2/O_2, 10 s) holey carbon grids (Quantifoil, R1.2/1.3, 300 mesh, Au). The grids were blotted for 5 s at 100% humidity and 4 °C with a Vitrobot Mark IV (Thermo Fisher Scientific) and plunge-frozen into liquid ethane cooled by liquid nitrogen. The blotted grids were stored in liquid nitrogen until imaging.

The data sets were collected on a Titan Krios cryo-electron microscope (FEI) operated at 300 kV equipped with a Gatan K2 Summit direct detection camera using the automated image acquisition software SerialEM[2] in counting mode. For apo form, movies were recorded with SA29, 000× magnification yielding a pixel size of 1.01 Å at University of Science and Technology of China. The total dose of 56 $e^{-}/Å^2$ was fractionated to 32 frames with 0.18 s per frame. Nominal defocus values ranged from -1.0 to -1.4 μm. For EVP-bound form and EVP/PNU-bound form, movies were recorded with SA29, 000× magnification yielding a pixel size of 1.014 Å at Zhejiang University. The total dose of 62 $e^{-}/Å^2$ was fractionated to 40 frames with 0.2 s per frame. Nominal defocus values ranged from -1.1 to -1.3 μm. Data sets of the apo form, EVP-bound form and EVP/PNU-bound form included 2,047, 2,800 and 2,948 movies, respectively.

Cryo-EM data processing

All data sets were processed similarly in relion3.1[3] and cryoSPARC2[4]. Dose-fractionated image stacks were subjected to beam-induced motion correction and dose-weighting using UCSF MotionCor2[5]. Contrast transfer function parameters were estimated with Gctf[6]. For particle picking, around 2,000 particles were picked manually to generate templates for auto-picking in relion3.1. Auto-picked particles were extracted in relion3.1, then imported into cryoSPARC2 for subsequent 2D classification. Particles from well-defined 2D averages were selected and combined for 3D

classification. An ab initio 3D reconstruction from the 2D average particles was generated in cryoSPARC2. The initial model was then used as a reference for 3D classification by heterogenous refinement without symmetry imposed. A selected class with continuous density for all transmembrane helices was used to perform further 3D classification by heterogenous refinement with C5 symmetry imposed in cryoCPARC2. Particles in good class were subjected in non-uniform refinement and produced the final 3D reconstruction. The overall resolutions were estimated by applying a soft mask excluding detergent micelle and the gold-standard Fourier shell correlation (FSC) using the 0.143 criterion. Local resolution was determined using cryoCPARC2.

Model building, refinement and validation

Atomic models were built in the software Coot[7]. For the model of apo-α7, the ECD was built using the crystal structure of alpha7-AChBP chimera (PDB code: 3SQ9) as a reference, while the TMD and ICD were built de novo. Every residue was manually examined. The initial model was subjected to iterative manual rebuilding in Coot and real-space refinement in PHENIX[8]. The final model was validated using the module "comprehensive validation (cryo-EM)" in PHENIX[9]. The residues from the N-terminal signal peptide (1-22) and the linker between helices MX and MA (347-431) were not built due to the lack of corresponding densities. N-acetylglucosamine moieties were built to linking to Asn46 and Asn133 sites, respectively, based on the corresponding densities. A cholesterol mimic was assigned according to the extra density nestled the M3, M4 and MX helices in each subunit. To build the models of α7/EVP and α7/EVP/PNU complex, the model of apo form was docked into the individual EM density maps and further subjected to manual rebuilding, real-space refinement and validation. All the figures were prepared using UCSF Chimera[10].

Electrophysiology experiments

Chinese hamster ovary (CHO) cells were cultured in DMEM/F12 medium (Gibco) supplemented with 10% fetal bovine serum (FBS), 100 U/mL penicillin, and 100 U/mL

streptomycin at 37 °C in a 5% CO_2 incubator. For each transfection of a 24-well-plate well, 0.6 μg of plasmid encoding $\alpha 7_{EM}$ and 0.6 μg of plasmid encoding NACHO, or 0.6 μg of plasmid encoding α7, 0.6 μg of plasmid encoding NACHO and 0.2 μg of plasmid encoding eGFP were diluted and mixed with lipofectamine 3000 (Invitrogen), then added to the cells. After incubation for 5 h, the cells were transferred to poly-L-lysine-coated slides to culture for another 16-24 h in fresh medium. They were then used for electrophysiological recording.

For whole-cell patch clamp recordings, the bath solution contained 150 mM NaCl, 4 mM KCl, 2 mM $CaCl_2$, 1 mM $MgCl_2$, and 10 mM HEPES (pH 7.4, ~300 mOsm). The electrodes were pulled from thick-walled borosilicate glass capillaries with filaments (1.5 mm diameter, Sutter Instruments) on a four-stage puller (P-1000, Sutter Instruments) and had resistances of 2.5-4.5 MΩ when filled with intracellular solution containing 140 mM KCl, 10 mM NaCl, 2 mM $CaCl_2$, 1 mM $MgCl_2$, 5 mM EGTA, and 10 mM HEPES (pH 7.4, ~297 mOsm). All chemicals were obtained from Sigma. Experiments were performed at room temperature with an EPC-10 amplifier (HEKA Electronic) using the data acquisition software PatchMaster. Membrane potential was held at -70 mV in all experiments. Currents were recorded by exchanging the bath solution with the solution containing agonist or PAM through a Y-tube perfusion system. Agonists were applied by rapidly exchanging. The PAM was applied 30 s before the agonist. The α7 channel activated by agonist perfused at least every 5 min in flow rate of 4 mL/min to allow for a total exchange of local solution without agonist and a complete recovery of the channels from desensitization. The data were analyzed with Clampfit.

Intracellular calcium release measurements

HEK293T cells were co-transfected with plasmids of α7 (or mutants) and NACHO for 24 h and plated into flat-bottom 96-well plates (poly-d-lysine precoated) with 100 μL per well (100,000 cells per well). Intracellular calcium measurements were performed with the fluo-4 (Invitrogen) according to the manufacturer's protocols using the fresh

calcium buffer (HBSS buffer supplemented with 0.1% BSA, 2.5 mM probenecid, 3 mM $CaCl_2$, 1 mM $MgCl_2$, pH 7.4). The cells were loaded with 50 μL of the dye per well and incubated at 37 °C for at least 45 min. After a wash, the cells were incubated with 80 μL of the calcium buffer. Fluorescence measurements were recorded simultaneously from all the wells by a FLIPR. Plates were illuminated at 488 nm and fluorescence emission was recorded at 540 nm. Baseline fluorescence was typically measured for the first 10 s, after which 40 μL PNU-120596 with 4× final concentration was added to the cell plate and incubated for 5 min. The fluorescence intensity was captured every 1 s for the first 1 min followed by every 5 s for an additional 4 min. This procedure was followed by the addition of 40 μL agonist with 4× final concentration and readings were taken for a period of 5-7 min as described above.

REFERENCES

1 Gu, S. *et al.* Brain alpha7 Nicotinic Acetylcholine Receptor Assembly Requires NACHO. *Neuron* **89**, 948-955 (2016).

2 Mastronarde, D. N. SerialEM: A Program for Automated Tilt Series Acquisition on Tecnai Microscopes Using Prediction of Specimen Position. *Microscopy and microanalysis* **9**, 1182-1183 (2003).

3 Zivanov, J. *et al.* New tools for automated high-resolution cryo-EM structure determination in RELION-3. *Elife* **7**, e42166 (2018).

4 Punjani, A., Rubinstein, J. L., Fleet, D. J. & Brubaker, M. A. cryoSPARC: algorithms for rapid unsupervised cryo-EM structure determination. *Nature Methods* **14**, 290-296 (2017).

5 Zheng, S. Q. *et al.* MotionCor2: anisotropic correction of beam-induced motion for improved cryo-electron microscopy. *Nature Methods* **14**, 331-332 (2017).

6 Zhang, K. Gctf: Real-time CTF determination and correction. *Journal of Structural Biology* **193**, 1-12 (2016).

7 Emsley, P., Lohkamp, B., Scott, W. G. & Cowtan, K. Features and development of Coot. *Acta crystallographica. Section D, Biological crystallography* **66**, 486-501 (2010).

8 Adams, P. D. *et al.* PHENIX: a comprehensive Python-based system for

macromolecular structure solution. *Acta crystallographica. Section D, Biological crystallography* **66**, 213-221 (2010).

9 Afonine, P. V. *et al.* New tools for the analysis and validation of cryo-EM maps and atomic models. *Acta crystallographica. Section D, Structural biology* **74**, 814-840 (2018).

10 Pettersen, E. F. *et al.* UCSF Chimera--a visualization system for exploratory research and analysis. *Journal of Computational Chemistry* **25**, 1605-1612 (2004).

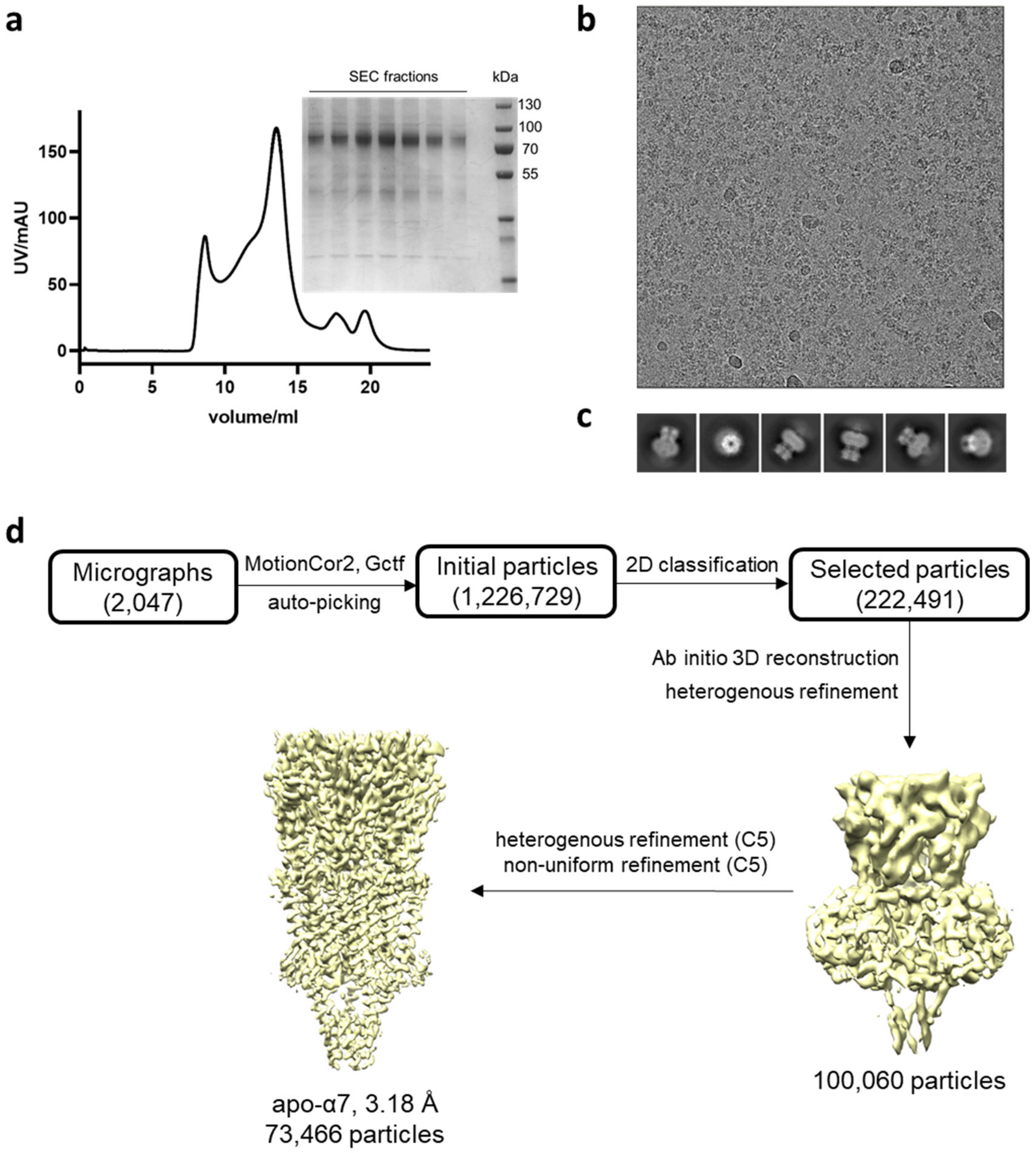

Fig. S1 Cryo-EM structure determination of the human α7 nicotinic receptor. a Size exclusion chromatogram of α7 and SDS-PAGE of main fractions collected from SEC. **b** A representative cryo-EM micrograph. **c** Representative 2D class averages. **d** Cryo-EM data processing flow chart of α7.

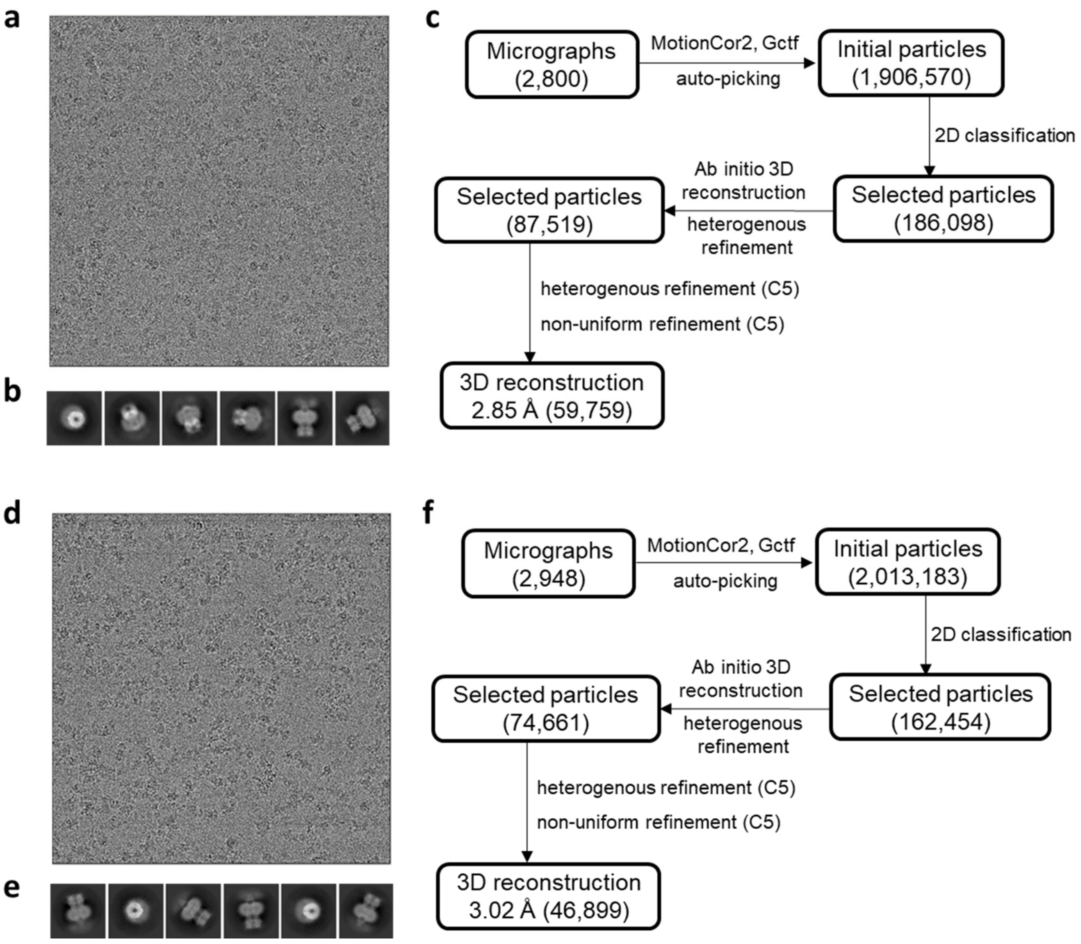

Fig. S2 Cryo-EM structure determination of the α7/EVP and α7/EVP/PNU complex. **a**, **b** A representative cryo-EM micrograph (**a**) and representative 2D class averages (**b**) of α7/EVP complex. **c** Cryo-EM data processing flow chart of α7/EVP complex. **d**, **e** A representative cryo-EM micrograph (**d**) and representative 2D class averages (**e**) of α7/EVP/PNU complex. **f** Cryo-EM data processing flow chart of α7/EVP/PNU complex.

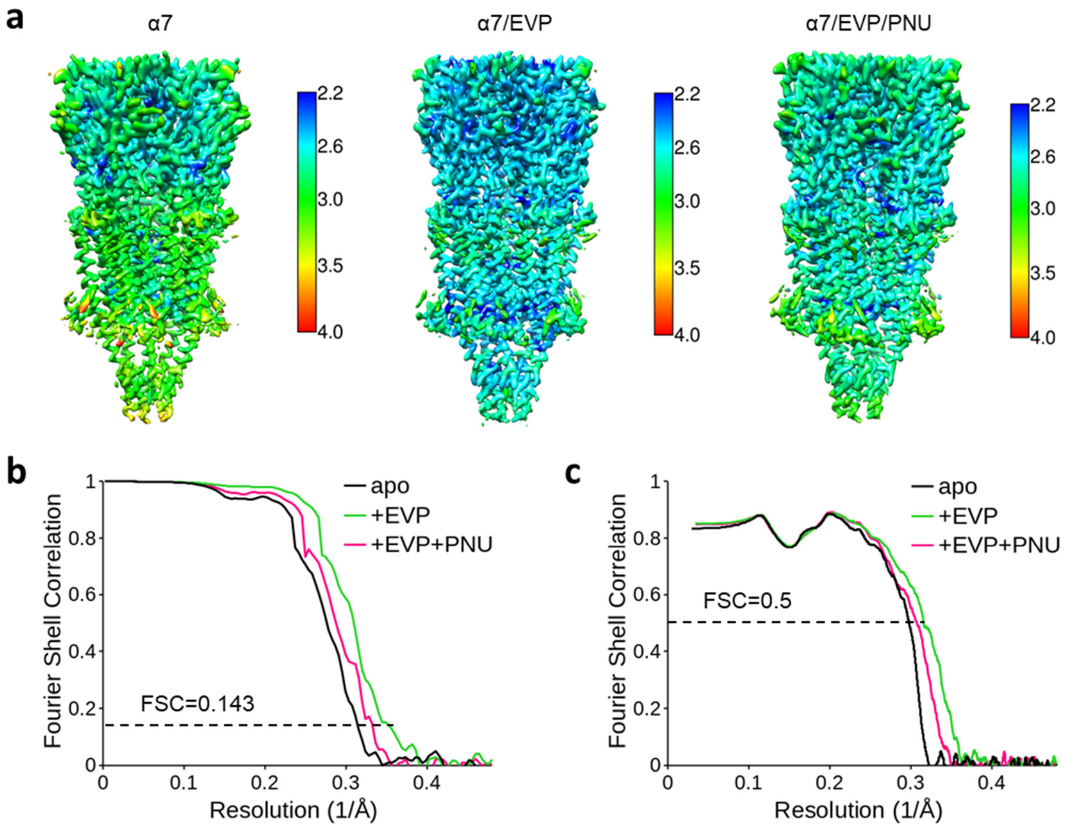

Fig. S3 Overall and local map resolution and global map-model agreement. a EM density of three α7 reconstructions colored according to local resolution estimate. **b** Gold-standard Fourier shell correlation curves from cryoSPARC. **c** Map-to-model Fourier shell correlation curves between each refined model and the corresponding maps.

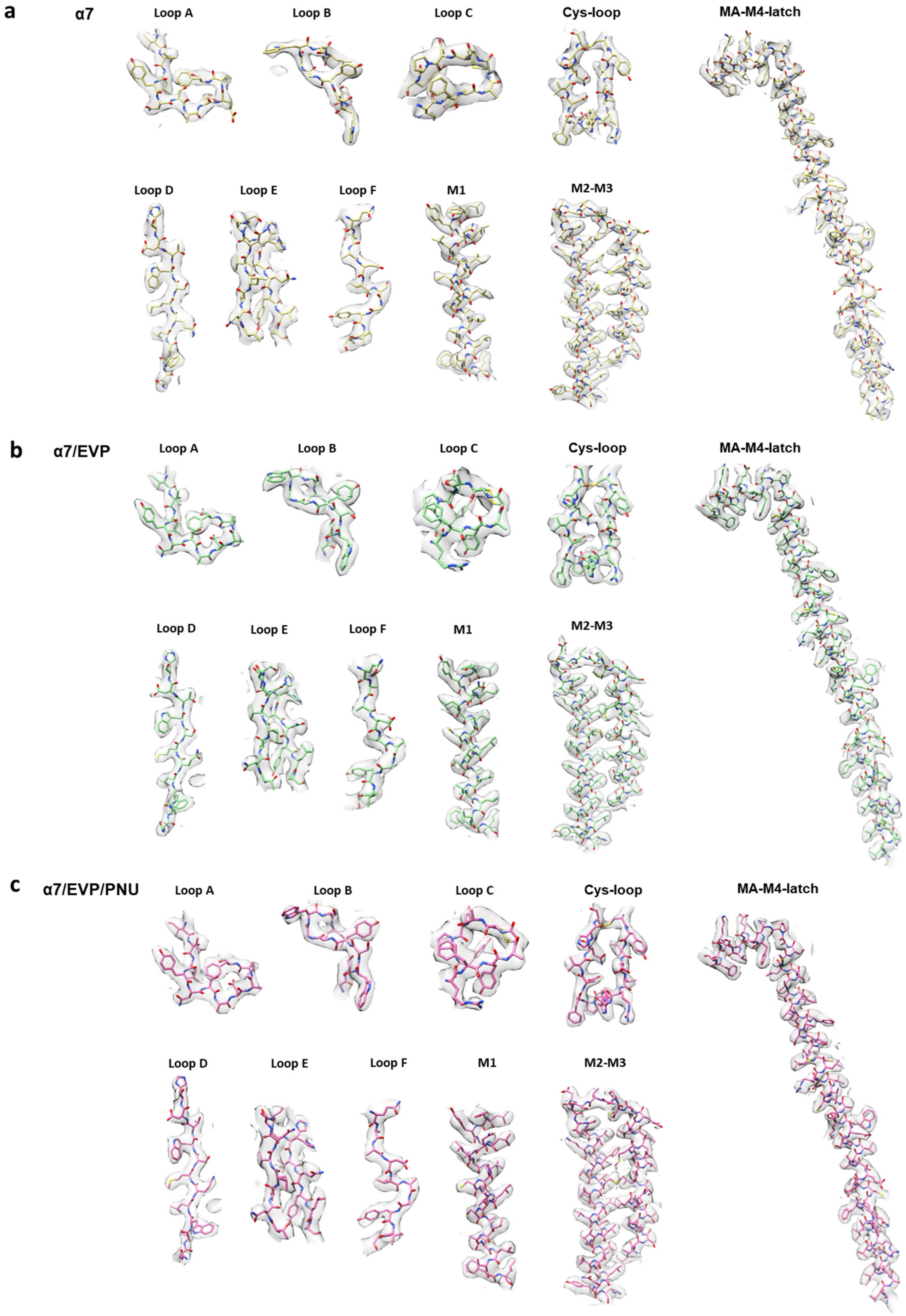

Fig. S4 Representative cryo-EM map quality. Representative densities and fitted atomic models of α7 (**a**), α7/EVP (**b**) and α7/EVP/PNU (**c**). Loops A, B, C, D, E, F, and Cys-loop in ECD, and α-helices M1, M2-M3, MA-M4-latch in TMD-ICD are shown.

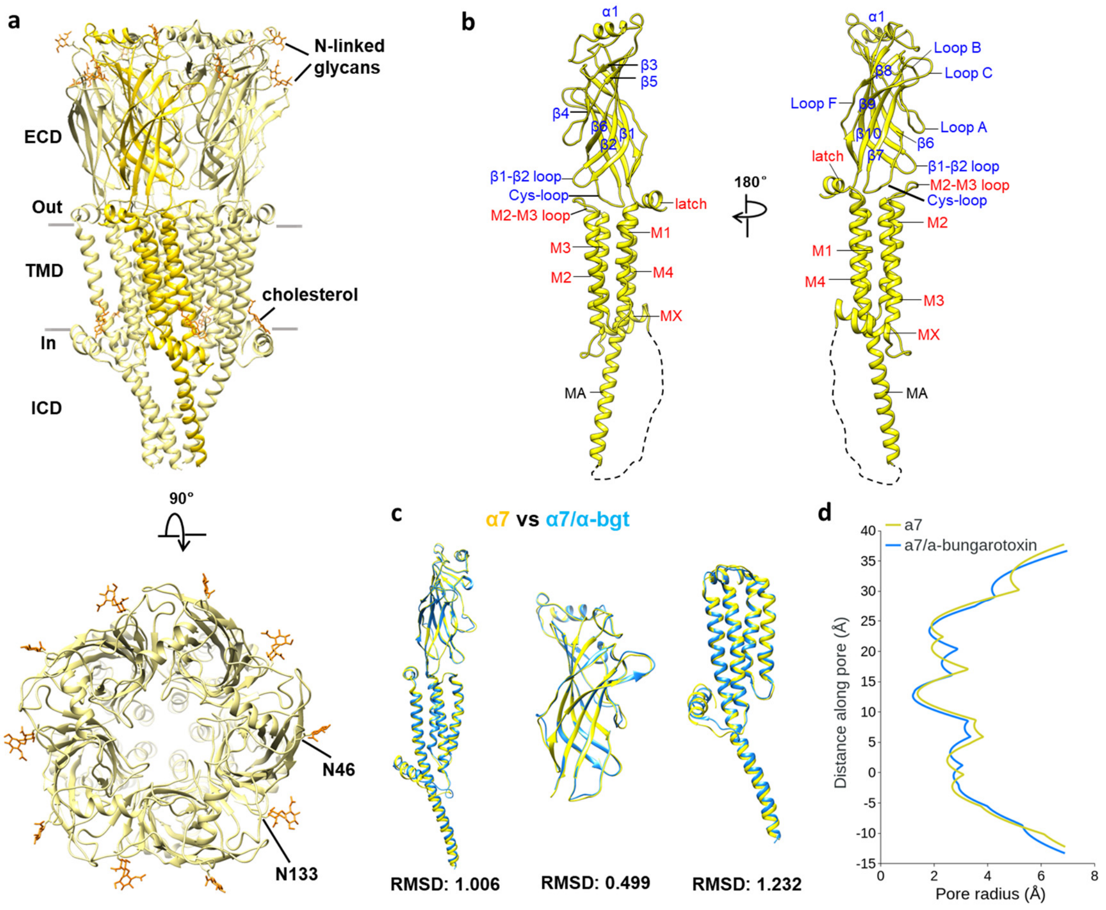

Fig. S5 Architecture of the human α7 nicotinic receptor. **a** Ribbon representation of α7 model viewed parallel to the membrane plane (top) from the extracellular space (bottom). The cell membrane is shown as grey lines. The Asn-linked carbohydrates and associated residues (Asn46 and Asn133), and cholesterol molecules are shown as sticks (brown). **b** Ribbon representation of a single subunit, viewed parallel to the membrane plane. The secondary structural elements and loops are labeled. Unmodeled residues linking helices MX and MA are represented as a dashed line. **c** Superimposition of a single subunit (left), ECD (middle) and TMD-ICD (right) from apo-form (yellow) and α-bgt-bound form (blue, PDB code 7koo). Their corresponding RMSDs between α-carbon pairs are shown underneath. **d** Plots of pore radius for α7 and α7/α-bgt along the pore axis. The α-carbon position of 0'-Lys (Lys261) is set to zero. Channel pore radius was calculated using the HOLE program.

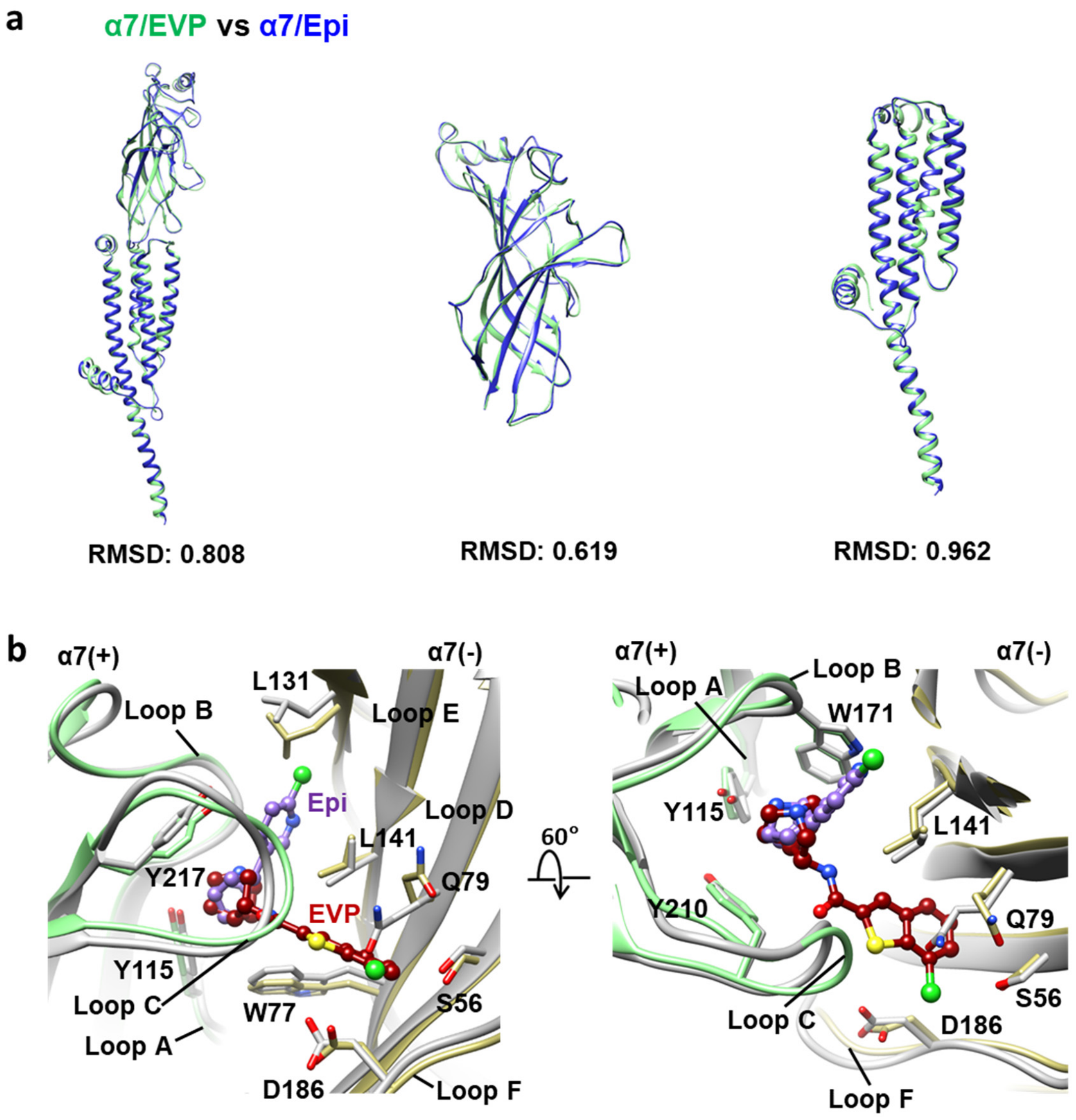

Fig. S6 Structural comparisons of α7/EVP and α7/Epibatidine. **a** Superimposition of a single subunit (left), ECD (middle) and TMD-ICD (right) from α7/EVP (green) and α7/Epibatidine (blue, PDB code 7koq). Their corresponding RMSDs between α-carbon pairs are shown underneath. **b** Comparison of ligand binding pockets in α7/EVP (green and yellow) with α7/Epibatidine (grey). EVP-6124, Epibatidine and interacting residues are shown as sticks.

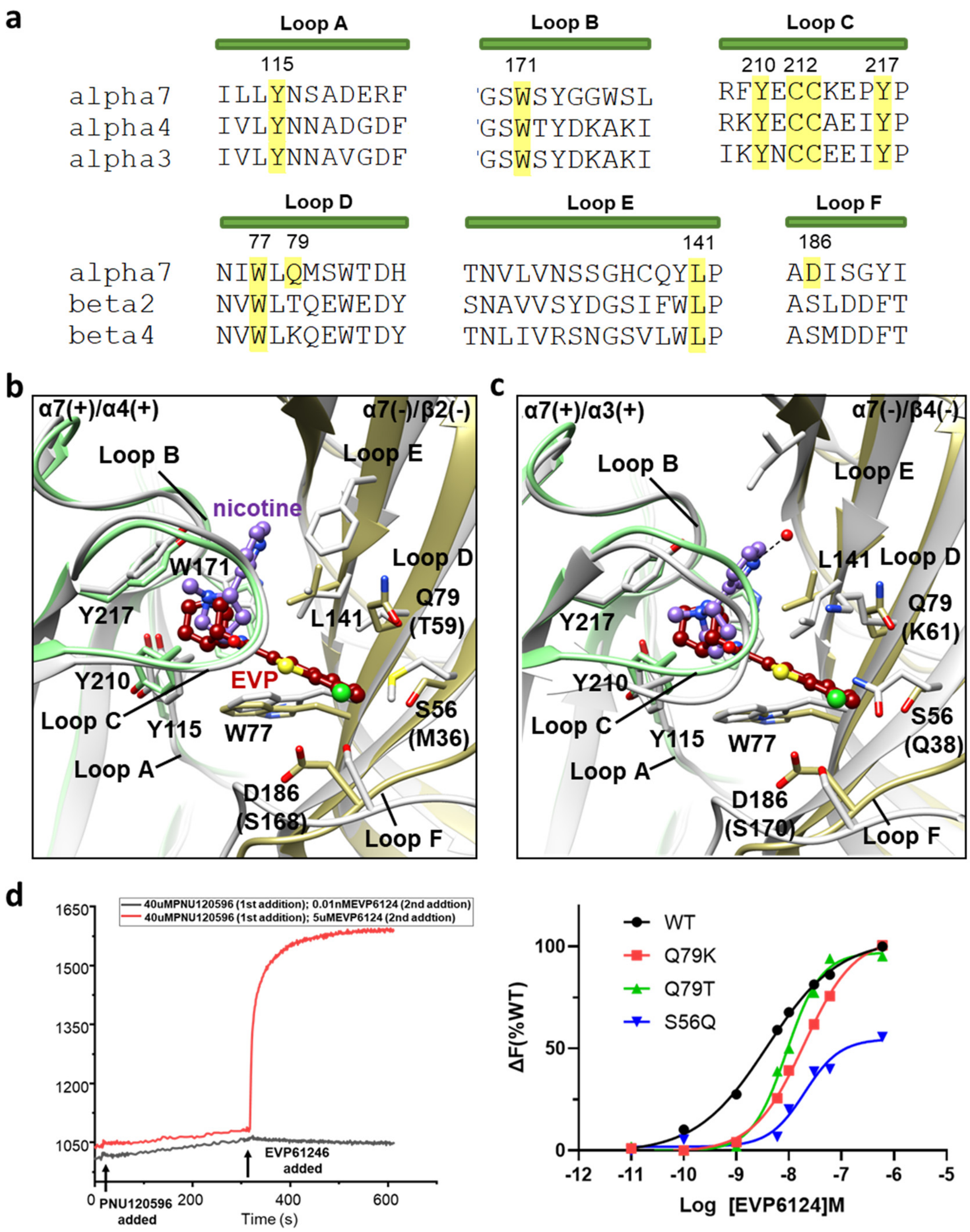

Fig. S7 Comparison of ligand binding pocket in α7/EVP with that in nicotine-bound α4β2 and α3β4 nicotinic receptors. **a** Sequence alignments of loops involved in EVP binding. Residues making interactions with EVP are labeled. **b**, **c** Overlay of α7/EVP (green and yellow) with α4β2 (grey, PDB code 6cnj) (**b**) and α3β4 (grey, PDB code 6pv7) (**c**), viewed parallel to the membrane plane. Residue numbering is for α7

and substitutions in subtypes are indicated in parentheses. **d** α7 response to EVP was evaluated using FLIPR and calcium flux measurements. (Left) Representative examples of changes in fluorescence detected in transfected cells with different concentrations of EVP. Two-step addition protocol illustrates the activity of an agonist in the second addition. (Right) Concentration-response curve for EVP to α7 and the mutants in the presence of PNU.

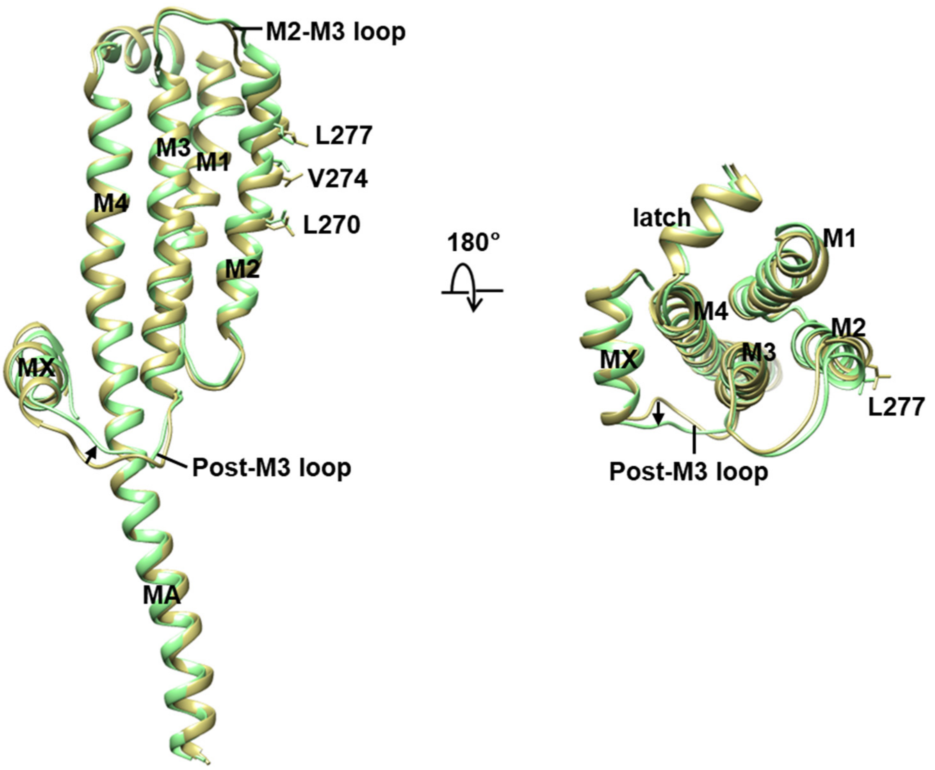

Fig. S8 Structural comparisons of TMD-ICD in α7 and α7/EVP. Superimposition of TMD and ICD in apo (yellow) and EVP-bound (green) α7 nicotinic receptor, viewed parallel to the membrane plane (left) and from the extracellular space (right). The residues lining the pore in the upper half of M2 are shown in sticks.

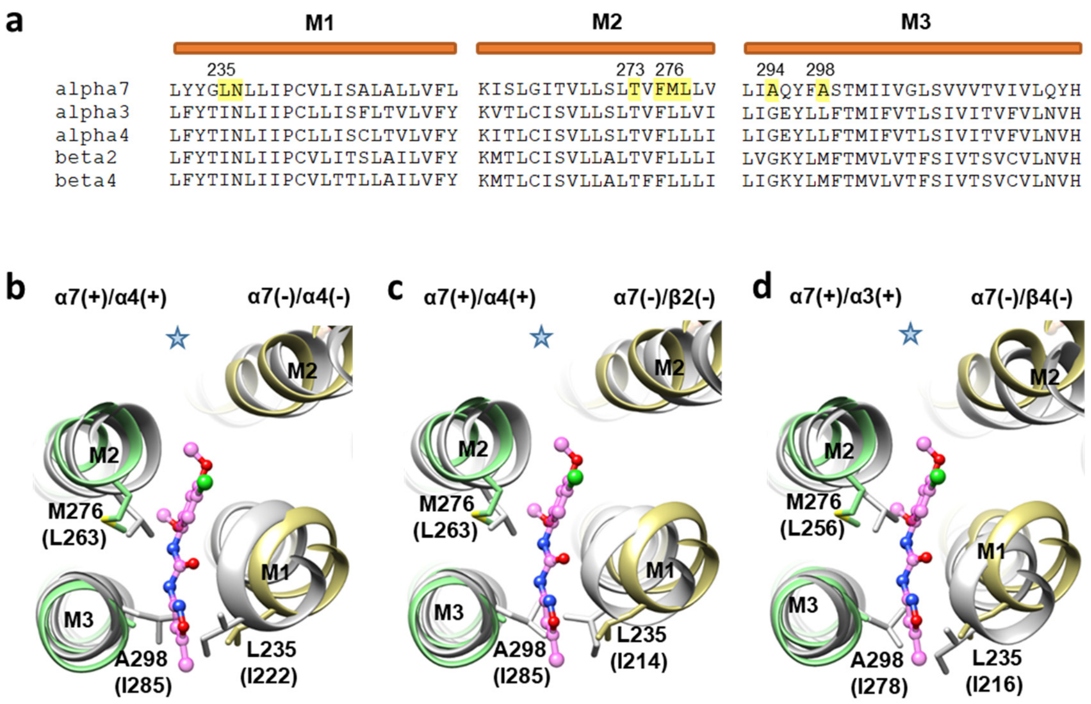

Fig. S9 Structural comparisons of PAM binding pocket in α7 with that in α4β2 and α3β4 nicotinic receptors. a Sequence alignments of M1-M3 helices. Residues making interactions with PNU are labeled. **b**-**d** Superposition of the TMDs in EVP/PNU-bound α7 (green and yellow), nicotine-bound α4β2 (grey, 3α2β, PDB code 6cnk, and 2α3β, PDB code 6cnj) (**b**, **c**) and α3β4 (PDB code 6pv7) (**d**) using the TMD from the (+)-subunit, viewed from the extracellular space. Residue numbering is for α7 and substitutions in subtypes are indicated in parentheses. The star symbol represents the pore axis.

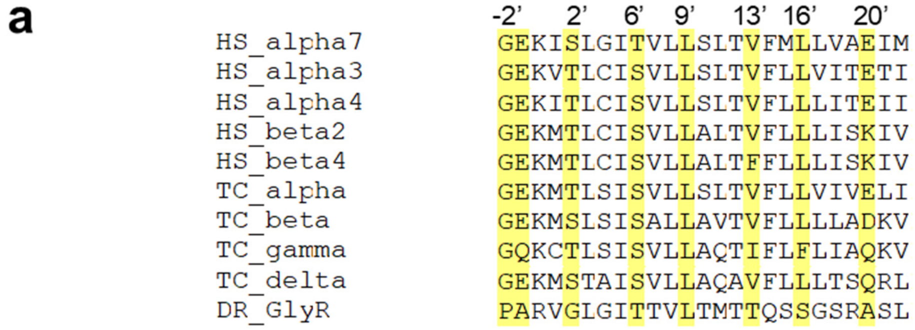

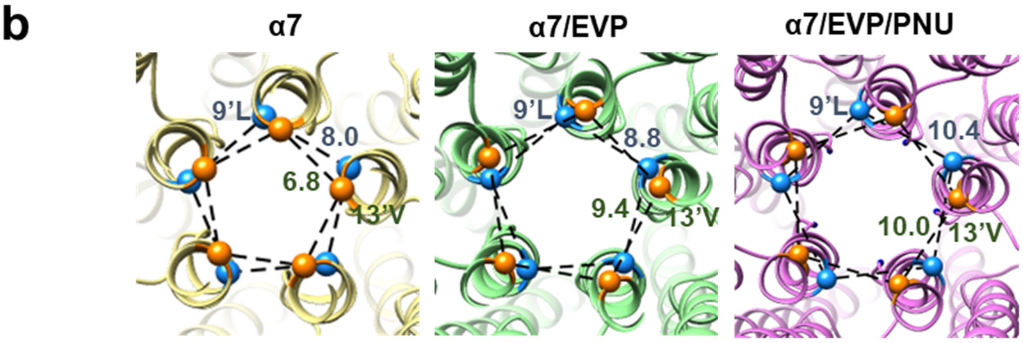

	16'Leu	13'Val	9'Leu	-2'Gly
α7	8.2	6.8	8.0	6.5
α7/EVP	10.4	9.4	8.8	6.6
α7/EVP/PNU	11.0	10.0	10.4	6.6

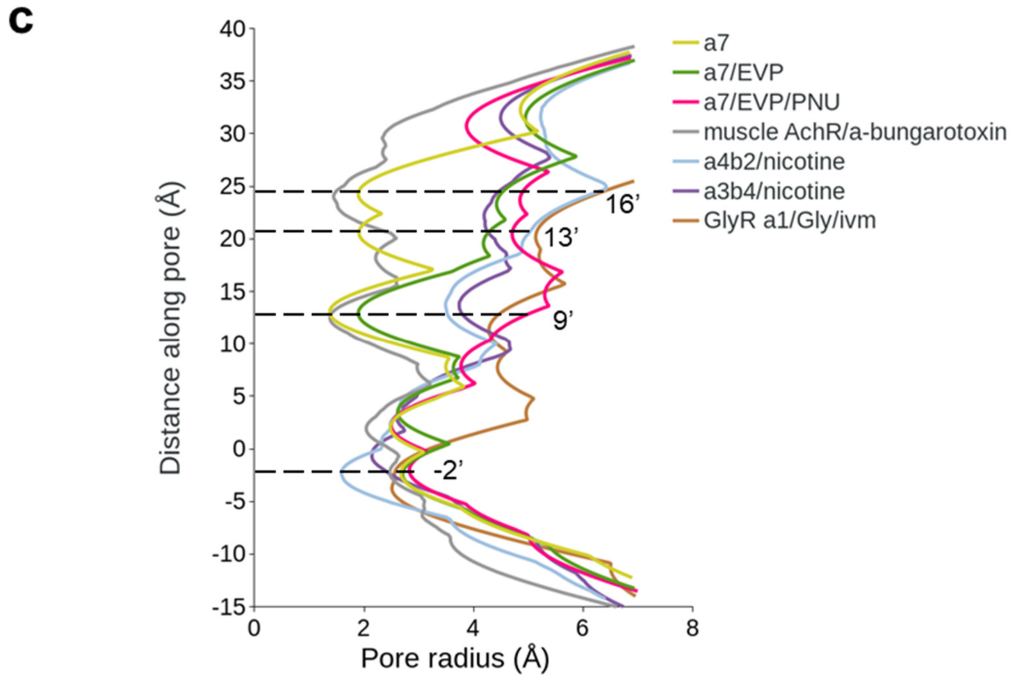

Fig. S10 Channel pore. a Sequence alignment of the M2 α-helices. Residues lining the pore are highlighted in yellow. **b** Pore constriction sites at 13'Val and 9'Leu are viewed

from the extracellular space, with their α-carbon in brown and blue spheres, respectively. Distances between the adjacent α-carbon of 16'Leu, 13'Val, 9'Leu and -2'Gly are shown in angstroms. **c** Plots of pore radius for receptors along the pore axis. The α-carbon position of 0'-Lys (Lys261) is set to zero. Channel pore radius was calculated using the HOLE program.

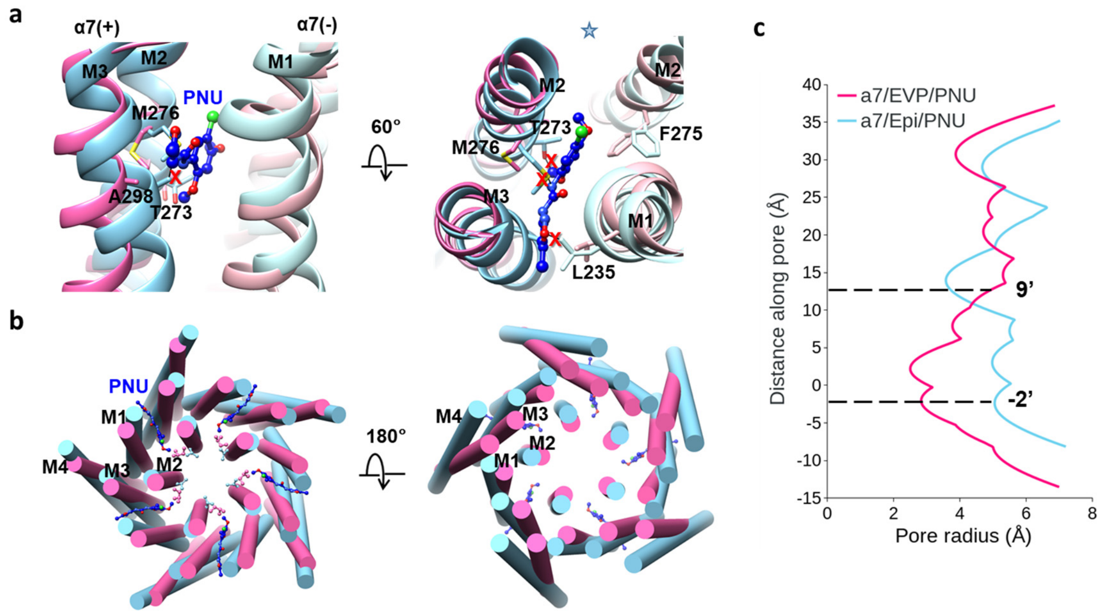

Fig. S11 Structural comparisons of α7/EVP/PNU and α7/Epibatidine/PNU. a Comparison of PAM binding pocket in α7/EVP/PNU (pink) with α7/Epibatidine/PNU (blue, PDB code 7kox). PNU and interacting residues in α7/EVP/PNU are shown as sticks. Potential clashes between PNU and α7/Epibatidine/PNU structure are indicated with red cross. The star symbol represents the pore axis. **b** Comparison of TMDs in α7/EVP/PNU (pink) with α7/Epibatidine/PNU (blue). Side chains of Leu270 are shown in balls and sticks. **c** Plots of pore radius for α7/EVP/PNU and α7/Epibatidine/PNU along the pore axis. The α-carbon position of 0'Lys (Lys261) is set to zero. Channel pore radius was calculated using the HOLE program.

Supplementary information, Table S1

Statistics of cryo-EM data collection, 3D reconstruction and model refinement.

	α7 (EMDB-31168) (PDB 7EKI)	α7/EVP (EMDB-31172) (PDB 7EKP)	α7/EVP/PNU (EMDB-31176) (PDB 7EKT)
**Data collection and processing**			
Magnification	29,000	29,000	29,000
Voltage (kV)	300	300	300
Electron exposure ($e^-/Å^2$)	56	62	62
Defocus range (μm)	-1.0~-1.4	-1.1~-1.3	-1.1~-1.3
Pixel size (Å)	1.01	1.014	1.014
Symmetry imposed	C5	C5	C5
Initial particle images (no.)	1,226,729	1,906,570	2,013,183
Final particle images (no.)	73,466	59,759	46,899
Map resolution (Å)	3.18	2.85	3.02
FSC threshold	0.143	0.143	0.143
**Refinement**			
Model resolution (Å)	3.35	3.17	3.24
FSC threshold	0.5	0.5	0.5
Map sharpening *B* factor ($Å^2$)	-129.0	-115.6	-119.4
Model composition			
Non-hydrogen atoms	16,005	16,115	16,195
Protein residues	1975	1980	1980
Ligands		5 (EVP)	5 (EVP)<br>5 (PNU)
B factors ($Å^2$)			
Protein	81.26	73.85	87.21
Ligand		67.16 (EVP)	73.82 (EVP)<br>95.03 (PNU)
R.m.s. deviations			
Bond lengths (Å)	0.003	0.003	0.003
Bond angles (°)	0.573	0.597	0.590
Validation			
MolProbity score	1.66	1.84	1.68
Clashscore	5.47	8.24	6.01
Poor rotamers (%)	0.88	0.88	0.30
Ramachandran plot			
Favored (%)	94.68	94.13	94.95
Allowed (%)	5.32	5.87	5.05
Disallowed (%)	0	0	0
